# From hemorrhage to apoptosis: understanding the devastating impact of ASFV on piglets

**DOI:** 10.1128/spectrum.02902-24

**Published:** 2025-07-11

**Authors:** Fanghua Xu, Ning Li, Yue Xue, Zhenru Wang, Zheng Fang, Hui An, Sidang Liu, Changjiang Weng, Li Huang, Gang Wang

**Affiliations:** 1 Shandong Provincial Key Laboratory of Zoonoses, College of Veterinary Medicine, Shandong Agricultural University, Taian, China; 2 State Key Laboratory for Animal Disease Control and Prevention, Harbin Veterinary Research Institute, Chinese Academy of Agricultural Sciences, Harbin, China; National Microbiology Laboratory, Winnipeg, Manitoba, Canada

**Keywords:** ASFV, multiple organ lesions, pathogenicity, apoptosis, piglets

## Abstract

African swine fever (ASF) is a devastating swine infectious disease that poses a serious threat to the global swine industry. Since ASF was first reported in China in 2018, this disease has caused significant economic losses in China. To characterize the histopathological changes induced by ASF virus (ASFV) in pigs and elucidate its pathogenic mechanism, this study used the ASFV HLJ/18 strain to infect specific pathogen-free piglets. The thymus, bone marrow, spleen, tonsil, lymph node, heart, liver, lung, and kidney were collected within the tenth day post-infection for histopathology, immunohistochemistry (IHC), TdT-mediated dUTP nick-end labeling, and Martius Scarlet Blue staining tests. The results showed that ASFV was widely distributed in various organs. Hemorrhage, cellular degeneration, and necrosis were observed in all examined organs, accompanied by a significant loss of lymphocytes in the immune organs, along with a large number of necrotic and apoptotic cells. Indirect immunofluorescence assay and IHC further showed that ASFV can infect monocytes and macrophages, epithelial cells, and hepatocytes. Apoptosis was prominently observed, particularly in lymphocytes within the immune organs, but it is worth noting that ASFV induced apoptosis in many different cell types in addition to lymphocytes. Furthermore, fibrin was observed in the blood vessels of multiple tissues, which may be associated with disseminated intravascular coagulation. These results demonstrate that the ASFV HLJ/18 strain was highly pathogenic to piglets, leading to severe pathological damage. This study provides valuable insights for deeply analyzing the pathogenicity mechanisms of ASFV.

**IMPORTANCE:**

Since African swine fever (ASF) first appeared in China in 2018, it has seriously jeopardized the healthy development of China's swine industry and caused huge economic losses. The characteristic of ASF is extensive hemorrhage in the organs, accompanied by immune suppression in the infected pigs. This study examined the damage caused by the virus and related mechanisms in histopathology and immunopathology. ASFV targets the macrophages, induces massive cell necrosis, and the formation of vascular microthrombi in multiple systems, leading to severe tissue damage and high mortality. Additionally, apoptosis and necrosis occurring in immune organs lead to an imbalance in the number and proportion of lymphocytes, further developing immune suppression. In conclusion, this study provides comprehensive insight into the immunopathology of ASFV infection.

## INTRODUCTION

African swine fever (ASF) is a highly contagious and severe infectious disease caused by ASF virus (ASFV). The World Organisation for Animal Health (WOAH) has classified it as a class A disease. ASF has had a huge impact on the global pig industry (1). It is characterized by high morbidity and mortality and rapid transmission. Infected pigs exhibit distinct clinical symptoms, including hyperpyrexia, depression, anorexia, generalized congestion, and cyanosis (2). ASF was first detected in Kenya in 1921 and has since severely affected the pig industry in many sub-Saharan African countries, some Eastern and Central European countries, and Southeast and East Asia (3). ASF first appeared in China in 2018 (4). Due to the lack of a safe and effective vaccine, the healthy development of China’s swine industry has been seriously jeopardized and caused huge economic losses.

ASFV belongs to the *Asfarviridae* family and is characterized as a large, enveloped, double-stranded DNA virus (5). The viral genome varies from 170 to 194 kb and encodes 150–200 proteins, including 68 structural proteins and more than 100 non-structural proteins (6). Currently, prevalent strains of ASFV have many genotypes with complex genetic variation. To date, a total of 24 genotypes of ASFV have been identified (7). In recent years, Georgia 2007-like strains of genotype II have become widespread throughout China, along with localized epidemics of genotype II virulence variants and genotype I low virulence strains (8, 9). The first genotype I/II recombinant virulent strain was also found in China in 2021 (10). The complexity of prevalent strains significantly increases the difficulty in preventing and controlling the disease.

Apoptosis is a unique cell death process (11) that plays a crucial role in clearing virus-infected cells to maintain tissue homeostasis (12). Infected cells initiate apoptosis to eliminate themselves and block viral replication and transmission, but some viruses can also trigger apoptosis to release and spread the progeny viruses (13, 14). ASFV infection and replication occur primarily in porcine macrophages (15). As the virus proliferates in the macrophages, the infected cells undergo necrosis and apoptosis. The virus is subsequently released into the bloodstream, lymphatic system, and various stromal tissues, leading to infections in different tissues and organs (16). Recent studies have found that apoptotic vesicles induced by ASFV promoted viral transmission (17).

The first Chinese strain of ASFV, named ASFV HLJ/2018, was isolated by the Harbin Veterinary Research Institute (18). This ASFV strain belongs to genotype II, serotype group 8, and has a high genetic homogeneity with the strain prevalent in Georgia (Georgia 2007/1). Results of previous animal experiments have shown that the ASFV HLJ/2018 strain has extremely high lethality and severe gross lesions of multiple organs in piglets (18). However, an in-depth understanding of histopathological damage and pathogenic characteristics of ASFV is still lacking. Due to the complexity of ASF prevalence in China, pathogenicity analysis using piglets infected with the original strain of ASFV HLJ/2018 can reveal the pathogenic characteristics of the disease more clearly. Therefore, the objective of this study was to analyze the histopathological lesions, viral distribution, and pathogenicity mechanism of ASFV HLJ/2018 in piglets.

## MATERIALS AND METHODS

### Virus and animals

The virus used in this study was ASFV HLJ/18. Virus propagation and 50% hemadsorption dose (HAD_50_) assay were performed as previously described (18). Briefly, primary porcine alveolar macrophage cells were used for virus propagation, and peripheral blood mononuclear cells were seeded in 96-well plates and infected in quintuplicate with 0.1 mL/well of 10-fold serially diluted supernatant. ASFV quantity was assessed by hemadsorption-induced rosette formation. The HAD_50_ was calculated at 7 dpi using the Reed-Muench method, with data presented as means from three independent experiments. HAD_50_ assay was performed to measure a titer of 10^3^ HAD_50_. Ten healthy specific pathogen-free (SPF) long white pigs with an initial weight of approximately 6 kg and an age of 8 weeks were used in this study. Samples of experimental pigs were collected, and the major endemic viruses (ASFV, classical swine fever virus, porcine circovirus [PCV] 2 and PCV3, pseudorabies virus, and porcine reproductive and respiratory syndrome virus) and their serum neutralizing antibodies were detected to ensure that the pigs were virologically and serologically negative.

### Animal experiments

The animal experimental protocol is described in detail by Huang et al. (19). Briefly, six SPF piglets were inoculated with ASFV HLJ/18 (10^3^ HAD_50_/piglet), and four SPF piglets were inoculated with phosphate-buffered saline (PBS). During necropsy, tissue samples including thymus, bone marrow, spleen, tonsil, lymph node, heart, liver, lung, and kidney were collected and fixed in 10% neutral buffered formalin for the following experiments.

### Antibodies and kits

The primary antibodies used in this study included mouse anti-porcine CD3ɛ coupled AF488 monoclonal antibody (Southern Biotech, USA), mouse anti-porcine CD3ɛ coupled SPRD monoclonal antibody (Southern Biotech), mouse anti-porcine CD14 coupled FITC (Bio-Rad, USA), rabbit anti-p-CK antibody (Bioss, China), and mouse anti-ASFV p72/p30 monoclonal antibody, which were prepared by immunizing mice with the purified recombinant p72 and p30 proteins. The secondary antibodies used in immunohistochemistry (IHC) and indirect immunofluorescence assay (IFA) were for goat anti-mouse IgG (H + L) (Beyotime, China), goat anti-mouse IgG-FITC, goat anti-mouse IgG-TRITC, and goat anti-rabbit IgG-FITC (Southern Biotech). The kits used in this study were *In Situ* Cell Death Detection Kit, POD Kit, TMR Red Kit (Roche, Germany), and MSB Kit (Solarbio, China).

### Histopathological and IHC analyses

All collected organs were fixed in 10% buffered formalin solution and embedded in paraffin, and the sections (3 µm in thickness) were prepared and stained with hematoxylin and eosin prior to observation by light microscopy. IHC staining was performed to identify the expression of ASFV p30 protein. Briefly, paraffin sections were treated with xylene deparaffinization, then heated in a microwave for 20 min using a 0.01 M sodium citrate buffer (pH 6.0), cooled to room temperature, and quenched for endogenous peroxidases in 3% H_2_O_2_ in methanol for 10 min. After blocking in 8% skimmed milk for 40 min at 37°C, the sections were stained with anti-ASFV p30 monoclonal antibody (1:20) at 4°C overnight, followed by incubation with a horseradish peroxidase (HRP)-labeled goat anti-mouse IgG (H + L) secondary antibody (1:50; Beyotime) for 1 h at room temperature. Finally, the sections were visualized by incubation with 3,3′-diaminobenzidine tetrahydrochloride (DAB) and counterstained with hematoxylin, and then viewed using a microscope (Nikon, Japan).

### Terminal deoxynucleotidyltransferase-mediated dUTP nick-end labeling (TUNEL)

DNA fragmentation was detected in 3 µm sections of paraffin-embedded tissues fixed in 10% buffered formalin. Paraffin sections were treated with xylene deparaffinization and quenched for endogenous peroxidases in 3% H_2_O_2_ in methanol for 10 min. The sections were permeabilized by incubation in 20 µg/mL proteinase K in PBS for 20 min. Afterward, the TUNEL method was used for the histochemical detection of apoptotic cells according to the manufacturer’s instructions. The samples were stained with TUNEL reagent (*In Situ* Cell Death Detection, POD; Roche) for 1 h at 37°C, with dropwise addition of HRP-labeled antibody against POD for 30 min at 37°C. Finally, the sections were visualized by incubation with DAB and counterstained with hematoxylin, and then viewed using a microscope (Nikon, Japan).

### Indirect IFA

Thymus from the challenge and control groups was selected for the indirect IFA. First, the tissues were dewaxed and hydrated, then heated in a microwave for 20 min using a 0.01 M sodium citrate buffer (pH 6.0). The sections were permeabilized by incubation in 20 µg/mL proteinase K in PBS for 20 min, after washing the tissues with TBST. A total of 20–40 µL anti-ASFV p72 monoclonal antibody (1:20) was used to cover the tissue at 4°C for 12 h, after which the tissues were washed with TBST and incubated with goat anti-mouse FITC or TRITC-conjugated antibodies (1:50; Southern Biotech) for 1 h at 37°C. Labeling of macrophages was performed using mouse anti-porcine CD14 coupled with FITC (Bio-Rad). Labeling of T-lymphocytes was conducted using mouse anti-porcine CD3ε coupled with AF488 monoclonal antibody (Southern Biotech), as well as mouse anti-porcine CD3ε coupled with SPRD monoclonal antibody (Southern Biotech). Labeling of epithelial cells was performed using rabbit anti-p-CK antibody (Bioss) and goat anti-rabbit IgG-FITC (Southern Biotech). Detection of apoptosis was performed using the *In Situ* Cell Death Detection Kit, TMR Red Kit (Roche) based on the protocol of Li et al. (20). Finally, the tissues were processed using DAPI (Sigma Aldrich, USA) and photographed using a confocal fluorescence microscope.

### Martius Scarlet Blue (MSB) assay

Staining of fibronectin in the organs and blood vessels was performed using the MSB Kit (Solarbio). Briefly, the sample preparation involved the following steps. First, the samples were dewaxed and treated in hypo solution. Next, the sections were immersed in Celestin blue solution for 3–5 min at room temperature, followed by a 3- to 5-min incubation in Mayer’s hematoxylin solution. Subsequently, the sections were treated sequentially with a 1% muriatic acid alcohol solution for 5–10 s, 95% alcohol for 5–10 s, Martius yellow solution for 2–3 min, crystal violet scarlet red solution for 10 min, and 1% phosphotungstic acid for 5 min. After treatment in aniline blue solution for 2–3 min, the sections were rinsed with 1% acetic acid. Finally, the presence of red color within the microvessels indicated the formation of microvascular thrombi.

## RESULTS

### ASFV infection caused lymphopenia and hemorrhage in multiple organs

**Fig 1:**
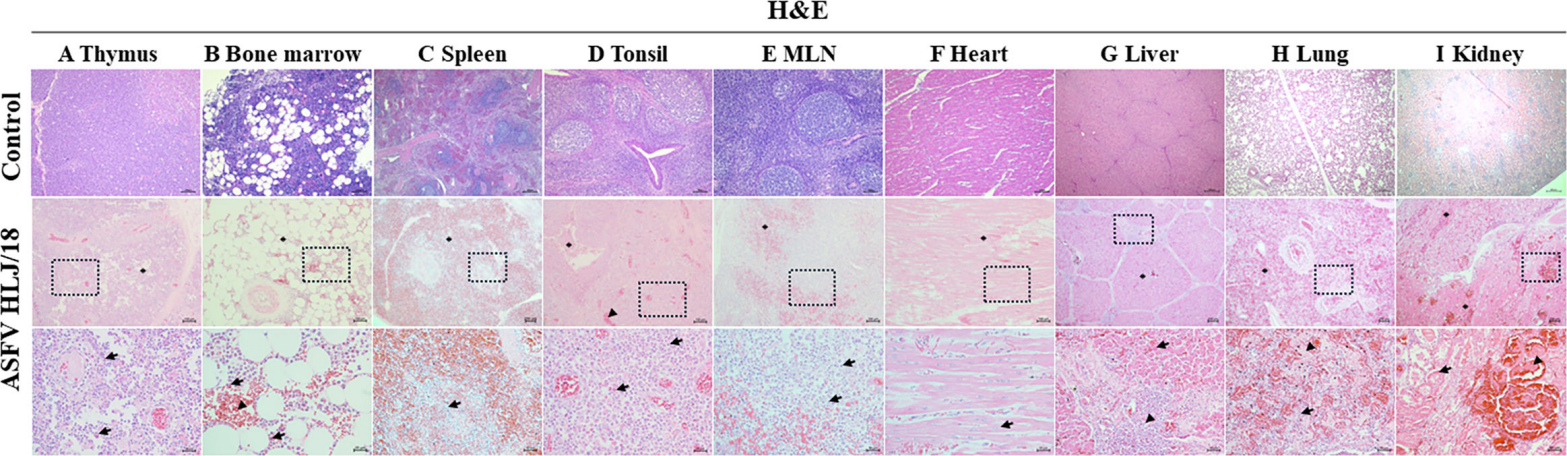
Histopathological features of the organs in the infected piglets compared with the control. (A) Thymus: massive reduction of thymic lymphocytes (⁕) and apoptotic state with cellular crumpling of multiple cells (arrows). (B) Bone marrow: massive decrease in immune cells (⁕), tissue hemorrhage (▲), and increase in apoptotic signature cells (arrows). (C) Spleen: red pulp full of erythrocytes (⁕), white pulp showing erythrocytes, apoptotic cells, and cellular debris (arrows). (D) Tonsil: detached crypt epithelial cells (⁕), congested blood vessels (▲), and sparse lymphocytes characteristic of apoptotic cells (arrows). (E) Mesenteric lymph nodes: hemorrhage in the region of the lymphatic sinus (⁕) and sparse lymphocytes characteristic of apoptotic cells (arrows). (F) Heart: myocardial fiber interstitial hemorrhage with inflammatory cell infiltration (⁕) and myocyte degeneration (arrows). (G) Liver: intrahepatic hemorrhage (⁕), portal area inflammatory cell infiltration (▲), and hepatocellular degeneration (arrows). (H) Lung: diffuse intra-alveolar interstitial intraproteinaceous edema and hemorrhage (⁕), intra-alveolar fibrin leakage (▲), and increased cell necrosis and apoptosis (arrows). (I) Kidney: diffuse multifocal hemorrhage in the renal cortex and medulla (⁕), detachment of tubular cells from the basement membrane (▲), and intraglomerular hyaline thrombi (arrows). Scale and magnification: 200 μm = 40×, 100 μm = 100×, 50 μm = 200×, and 25 μm = 400×.

In order to determine the pathogenicity of ASFV HLJ/18 in piglets, thymus, bone marrow, spleen, tonsils, mesenteric lymph nodes, heart, liver, lung, and kidney were collected and analyzed for histopathological changes within 10 dpi. Reduced thymus area in the infected group was observed, especially in the cortical portion, with a massive decrease in lymphocytes and cellular crumpling in multiple cells. These changes were consistent with morphological features of apoptosis (Fig. 1A). The bone marrow had a massive decrease in hematopoietic and immune progenitor cell aggregation regions, with severe hemorrhage (Fig. 1B). The white pulp of the spleen was reduced in area, with numerous erythrocytes accumulating in both the white and the red pulp, accompanied by a marked increase in cellular debris and necrotic or apoptotic cells (Fig. 1C). The tonsils showed crypt epithelial cell detachment, severe destruction of lymphoid tissue, and lymphocytopenia (Fig. 1D). Hemorrhage within the lymphatic sinusoids of the mesenteric lymph nodes, as well as lymphocytopenia, was observed as well (Fig. 1E). In addition, the analyses revealed interstitial hemorrhage, cardiomyocyte degeneration, and inflammatory cell infiltration in the heart (Fig. 1F). The liver showed intrahepatic hemorrhage, dilation of hepatic sinusoidal spaces, portal area proliferation with inflammatory cell infiltration, and diffuse hepatocyte degeneration (Fig. 1G). In the lungs, diffuse intra-alveolar interstitial intraproteinaceous edema was found, along with massive inflammatory cell infiltration and cells showing necrotic or apoptotic features (Fig. 1H). Finally, analyses revealed diffuse multifocal hemorrhage in the renal cortex and medulla, intra-glomerular hyaline thrombus, inflammatory cell infiltration, hyaline degeneration, and detachment of glomerulonephritic tubular cells from the basement membranes (Fig. 1I). No significant pathological changes of the various tissues and organs were found in the control group. The above results show that ASFV infection of piglets can cause severe pathological damage in multiple organs and lead to morphological features of apoptosis in a variety of cells.

### ASFV has a wide range of tissue tropism

**Fig 2:**
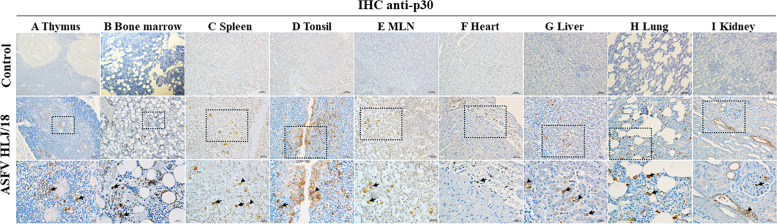
IHC detection of ASFV p30 reveals the distribution features in the organs of the infected piglets, as compared to the control. (A) Thymus: infection of cells with macrophage morphology (arrows). (B) Bone marrow: infection of monocytes (arrows). (C) Spleen: infections characterized by monocytes/macrophages (arrows) and epithelioid cells (▲). (D) Tonsil: infection of macrophages (arrows) and crypt epithelial cells (▲). (E) MLN: infection in lymphatic sinus mass macrophages (arrows). (F) Heart: infected macrophages located in the subepicardium (arrows). (G) Liver: infection of Kupffer cells (arrows) and hepatocytes (▲). (H) Lung: infection of macrophages (arrows). (I) Kidney: infection of macrophages (arrows) and renal tubular epithelial cells (▲). Scale and magnification: 200 μm = 40×, 100 μm = 100×, 50 μm = 200×, and 25 μm = 400×.

To clarify the ASFV distribution in organs and infected cell types of ASFV HLJ/18, IHC and IFA were used to localize the virus. The results showed that ASFV was widely distributed in the thymus, where the infected cells had the morphological characteristics of macrophages (Fig. 2A). Infection with monocytes was seen in the bone marrow (Fig. 2B). In the spleen, most of the infected cells had monocyte-macrophage features, but infection of epithelioid cells was also found (Fig. 2C). In the tonsils, in addition to macrophage infections, significant viral infections were observed in the crypt epithelial cells (Fig. 2D). There were more infected cells in the lymph nodes, concentrated in the lymphatic sinusoidal region (Fig. 2E). Infected macrophages were observed in the heart, located in the subepicardium (Fig. 2F). In the liver, infections of Kupffer cells and a small number of hepatocytes were observed (Fig. 2G). Macrophages were infected in the lungs (Fig. 2H). Finally, macrophages and renal tubular epithelial cells were infected in the kidney (Fig. 2I). The above results show that ASFV was widely distributed in multiple organs and that the predominantly infected cells had the morphological characteristics of macrophages.

**Fig 3:**
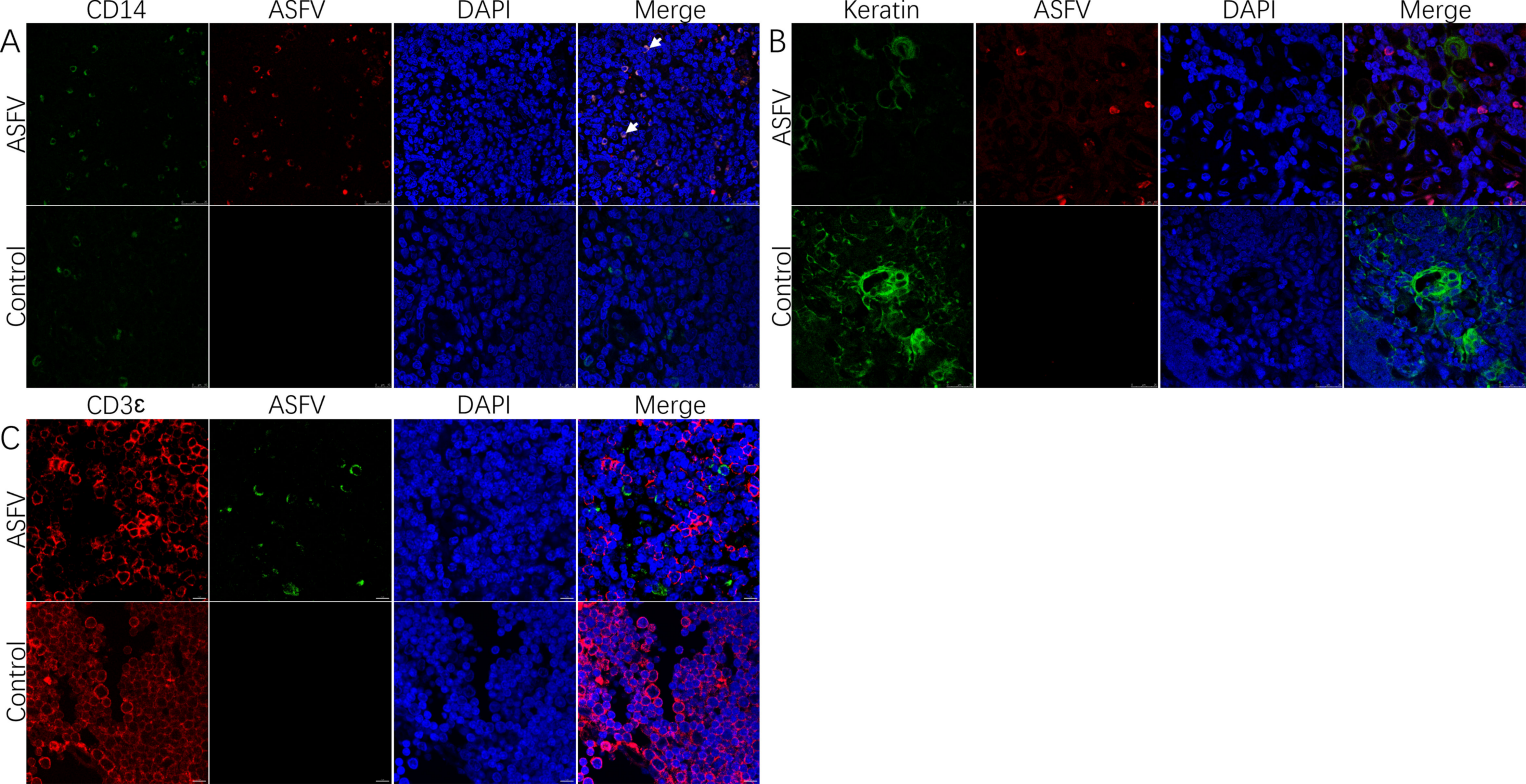
Identification of cell types that co-localize with ASFV p72 in the thymus. (A) Macrophages in the thymus, which co-localized markedly with the virus, were the predominantly infected cells (arrows). (B) Viral infection was not observed in the epithelial cells in the thymus. (C) Viral infection was not observed in the T-lymphocytes in the thymus.

In order to further clarify the type of infected cells, co-localization of different cells (epithelial cells, macrophages, and lymphocytes) within the thymus infected with ASFV was conducted using a dual fluorescent labeling test. The results showed that ASFV was able to co-localize with macrophages (Fig. 3A) but not epithelial cells or T-lymphocytes (Fig. 3B and C).

### ASFV induces apoptosis in a variety of cells

**Fig 4:**
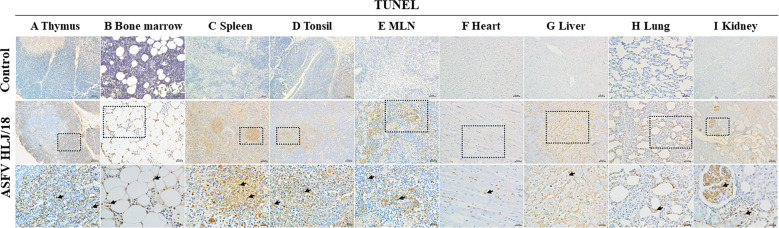
Apoptosis features of the organs in the infected piglets compared with the control. (A) Thymus: apoptotic cells mostly concentrated in the thymic cortex (arrows). (B) Bone marrow: abundant apoptotic cells (arrows). (C) Spleen: large number of apoptotic cells in the white pulp (arrows). (D) Tonsil: apoptosis mostly in the diffuse lymphoid tissue and lymphoid follicles under the crypts (arrows). (E) Mesenteric lymph nodes: apoptotic cells both inside and outside lymphoid follicles (arrows). (F) Heart: apoptosis of a small number of cardiomyocytes (arrows). (G) Liver: apoptosis of hepatocytes (arrows). (H) Lung: apoptosis of peri-alveolar cells (arrows). (I) Kidney: apoptosis of glomerular epithelial cells and tubular epithelial cells (arrows). Scale and magnification: 200 μm = 40×, 100 μm = 100×, 50 μm = 200×, and 25 μm = 400×.

In the histopathological analysis described above, the cells in the ASFV challenge group showed clear apoptotic features (Fig. 1A). Here, we further validated whether ASFV HLJ/18 induces apoptosis in tissue using the TUNEL method. The results showed that an abundant amount of apoptotic cells appeared in the thymus of the ASFV challenge group. Interestingly, apoptotic cells were mostly concentrated in the thymic cortex. Only normal individual apoptotic cells were seen in the control group (Fig. 4A). Apoptotic cells were widespread in the immune organs, not only in the thymus. In the bone marrow, apoptotic cells were abundant (Fig. 4B). There was massive apoptosis in the spleen, with a marked increase in the number of cell deaths observed in the white pulp (Fig. 4C). In the tonsils, apparent apoptosis was mostly seen in the diffuse lymphoid tissue and lymphoid follicles under the crypts (Fig. 4D). A large number of apoptotic cells was seen both inside and outside the lymphoid follicles in the mesenteric lymph nodes (Fig. 4E). The above experiments demonstrated that apoptotic cells were widely distributed in lymphoid tissues, and that, based on further cytomorphological analysis, apoptotic cells were mostly lymphocytes. Meanwhile, other types of apoptotic cells were found in the tested organs. Apoptosis of a small number of cardiomyocytes and hepatocytes was observed in the heart (Fig. 4F) and the liver (Fig. 4G); apoptosis of peri-alveolar cells in the lungs (Fig. 4H); and apoptosis of glomerular epithelial cells and tubular epithelial cells in the kidney (Fig. 4I).

**Fig 5:**
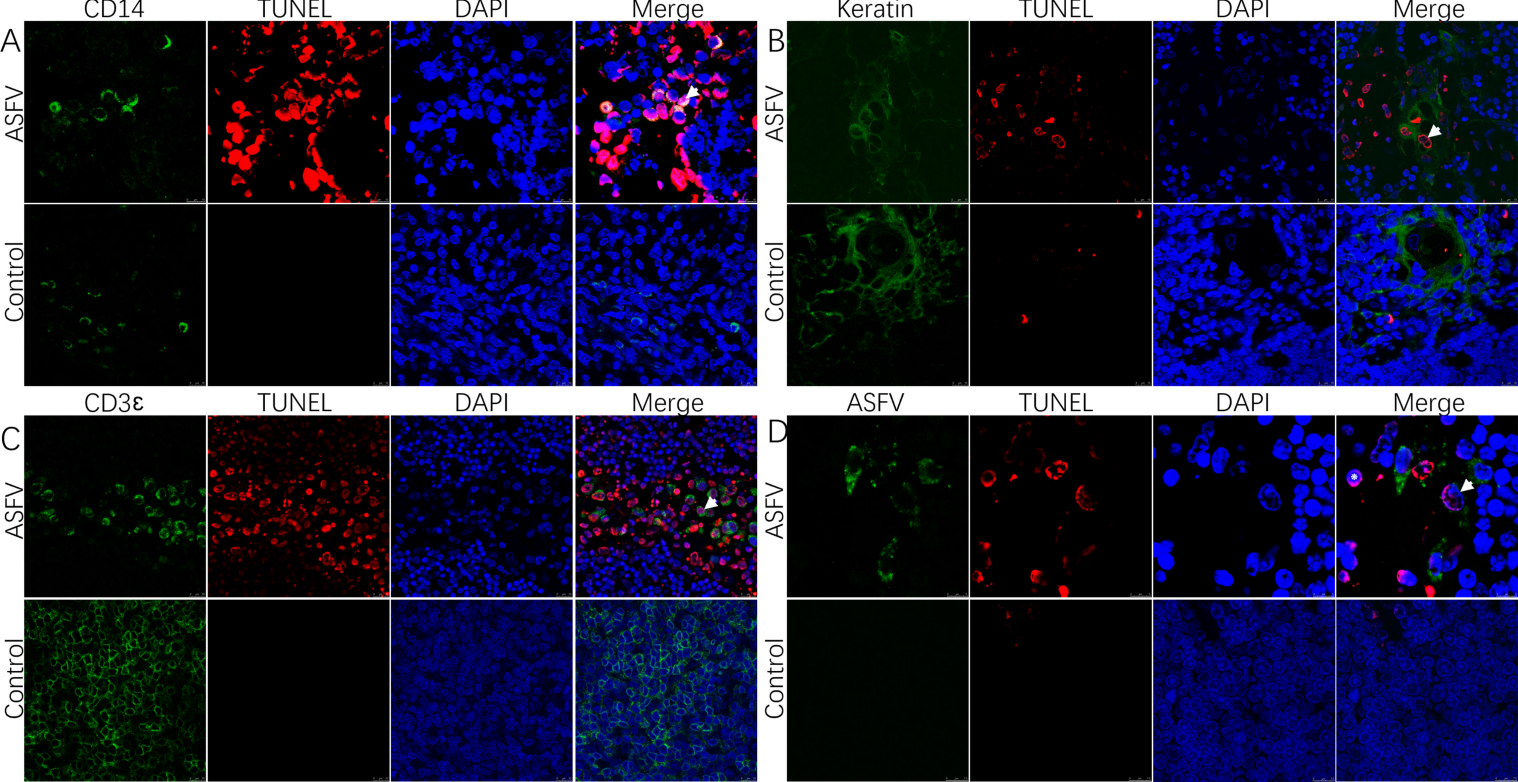
Identification of apoptotic cell types in the thymus and identification of apoptosis in infected cells. (A) Macrophages in the thymus, co-localized with apoptotic cells (arrows), suggesting apoptosis in macrophages. (B) Apoptosis observed in epithelial cells in the thymus (arrows). (C) Apoptosis observed in T-lymphocytes in the thymus (arrows). (D) Apoptosis in infected cells in the thymus (⁕) and in uninfected cells (arrows).

In order to further clarify the type of apoptotic cells, co-localization of different cells (epithelial cells, macrophages, and lymphocytes) within the thymus was conducted. The results showed that apoptosis occurred in macrophages (Fig. 5A), partial apoptosis in epithelial cells (Fig. 5B), and extensive apoptosis in T-lymphocytes (Fig. 5C). Notably, no viral infection of apoptotic cells was observed in ASFV and TUNEL double staining (Fig. 5D).

### ASFV causes multi-organ intravascular fibrin deposition and hyaline thrombi

**Fig 6:**
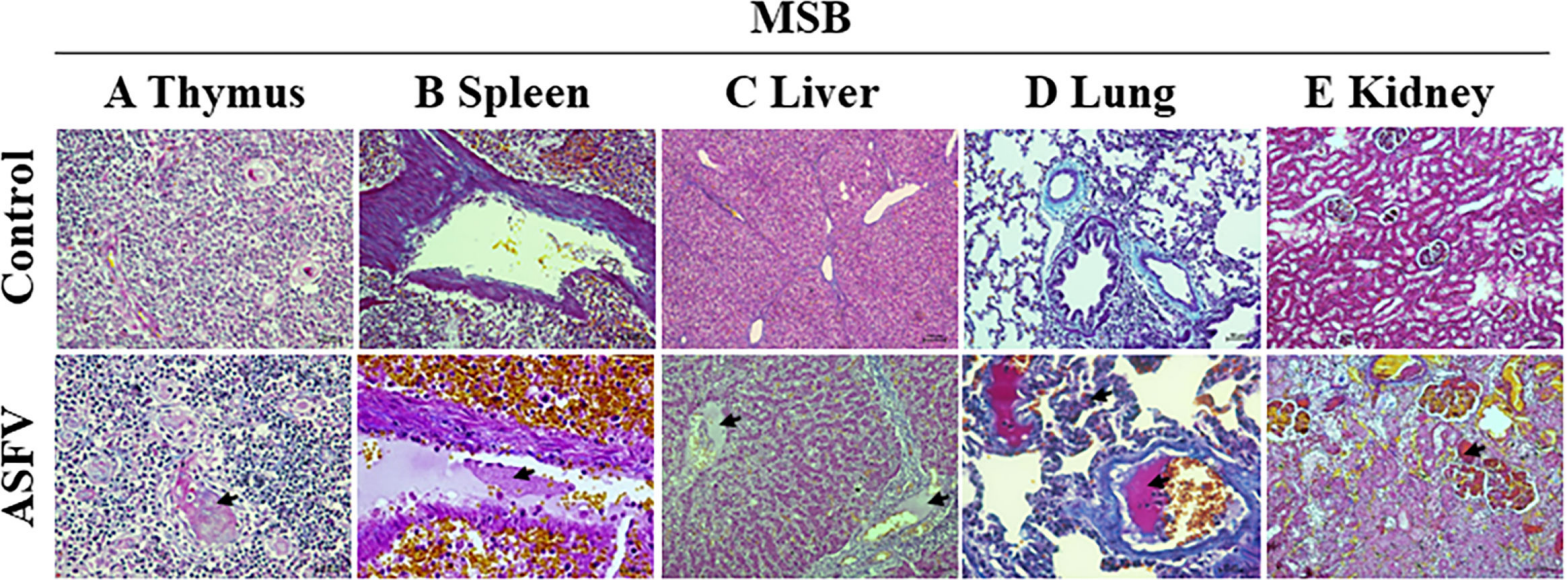
Fibrin distribution features of the organs in the infected piglets compared with the control. (A) Thymus: fibrin in the thymic vessels in the challenge group (arrows). (B) Spleen: fibrin deposits in the splenic vessels (arrows). (C) Liver: fibrin deposits in the central vein of the hepatic lobule (arrows). (D) Lung: formation of hyaline thrombi by massive fibrin deposition in small blood vessels and capillaries of the lungs (arrows). (E) Kidney: hyaline thrombi in glomerular capillaries (arrows). Scale and magnification: 200 μm = 40×, 100 μm = 100×, 50 μm = 200×, and 25 μm = 400×.

In histopathological staining observations, fibrin deposits and hyaline thrombi were seen in the tissues of the challenge group. It is suspected that this may be related to bleeding due to disseminated intravascular coagulation (DIC). Therefore, it was further verified by MSB staining. The results showed that the thymic vessels in the challenge group showed fibrin (Fig. 6A), as well as fibrin deposits in the splenic vessels (Fig. 6B) and the central vein of the hepatic lobule (Fig. 6C). Analyses also revealed the formation of hyaline thrombi by massive fibrin deposition in small blood vessels and capillaries of the lungs (Fig. 6D), as well as hyaline thrombi in glomerular capillaries (Fig. 6E). No fibrin was observed in any of the control vessels. The results suggested that ASFV infection caused extensive coagulation within the organ vasculature, where DIC lesions occurred.

## DISCUSSION

This study was carried out to gain a systematic understanding of the histopathological aspects in piglets experimentally infected with the genotype II ASFV HLJ/18 strain. The results filled in the gap in the histopathological lesions of the first Chinese strain of ASFV. The results showed that ASFV HLJ/18 was distributed in multiple organs of the infected piglets and caused severe pathological damage, massive reduction of immune cells, and hemorrhage in multiple tissues. Further analyses showed that apoptosis played a major role in the pathogenic process of the disease. In addition, DIC is a reason for tissue hemorrhage. In view of the complexity of the genetic variation and recombination of the ASFV gene (10, 21), this study provides a reference for the pathological diagnosis of ASFV. It also provides a model for the study of tissue pathogenicity of different ASFV strains.

The histopathological results of ASFV HLJ/18 showed no significant difference in the characteristics of the previously identified p72 genotype II strains (22–24). ASFV HLJ/18 exhibits severe histopathological damage in organs such as the spleen, lung, kidney, and liver, leading to organ failure (Fig. 1), which is consistent with injury characteristics of highly virulent strains (25). It is also accompanied by a decrease in lymphocytes in the immune organs, especially in the thymus. The above indicates that the strain is highly pathogenic and immunosuppressive. Studies have reported that mononuclear phagocyte system (MPS) cells are the main target cells of ASFV (26–28), while infections in other cell types have also been identified (29–31). ASFV HLJ/18 exhibited a predominant tropism for cells of MPS, consistent with its known pathogenesis. Notably, in this study, viral infection was also detected in epithelial cells and hepatocytes across multiple organs, as well as in unidentified cell populations, suggesting potential broader tissue tropism beyond classical MPS targets. However, there may be dissimilarities in the viral infection of other cell types, due to viral characteristics or the sensitivity of the assay. Multiorgan hemorrhage is a typical lesion of ASFV infection (32, 33). It has been shown that ASFV causes abnormalities in coagulation, which may result in consumptive coagulopathy and lead to bleeding (34, 35). Fibrin deposits and hyaline thrombi were found in the vessels of multiple organs in our study (Fig. 6), which is evidence of massive depletion of coagulant substances. Simultaneously, DIC represents a critical factor contributing to hemorrhage induced by ASFV infection (36), and our findings provide essential histopathological evidence supporting this mechanism. Notably, the above histological analyses showed similar results to CSFV and PRRSV, so a differential diagnosis is essential.

In order to investigate the cause of lymphocyte reduction in the immune organs, severe apoptosis was observed following ASFV infection, as judged by TUNEL staining and histopathological features of apoptosis. It has been reported that ASFV is capable of inducing apoptosis (29, 37, 38). Macrophages are the host cells of ASFV (26), and macrophage apoptosis occurs in ASFV infection (39). However, the extent of apoptosis varies between strains (40). In the present experiment, macrophage apoptosis was successfully localized in the thymus using a double IFA (Fig. 5A). This apoptosis is associated with the direct action of the virus. It has been shown that ASFV can encode a large number of proteins involved in the regulation of apoptosis in the host. Proteins such as pE199L and p54 promote apoptosis (41, 42), which facilitates the detachment of the virus from the host cell and enhances its infectivity. Recent studies have shown that ASFV accelerates its spread between macrophages through the medium of apoptotic vesicles (17).

In addition to apoptosis in virus-infected cells, apoptosis of uninfected cells was observed in the thymus by double staining with ASFV and TUNEL (Fig. 5D). Moreover, apoptosis of T-lymphocytes, epithelial cells, and other cells was observed, but they were not infected. It has been shown that the highly virulent ASFV Spain-70 strain induces apoptosis of lymphocytes in the thymus (38). This indirect induction of apoptosis in bystander cells can be found in other viruses, such as PRRSV, which predominantly infects macrophages and induces apoptosis in thymocytes, potentially leading to immunosuppression and compromised host defense mechanisms. Such an infection strategy contributes to the viral pathogenicity (43). It has been reported that ASFV can induce tissue damage through indirect mechanisms mediated by pro-inflammatory cytokines (44). ASFV infection produces cytokines such as TNF-α, IL-1, and IL-6 in high quantities (45). These cytokines play a key role in apoptosis, such as TNF-α’s initiation of an exogenous apoptotic pathway that directs cells toward programmed death (46). These cytokines are mostly produced by activated macrophages (47), so macrophage function is important in ASFV infection (48). In addition, apoptosis can also be induced by severe ischemia and hypoxia in tissue cells (49). Severe hemorrhage and circulatory disorders due to ASFV infection may also contribute to apoptosis. Finally, ASFV regulates host life activities through apoptosis, causing severe tissue damage as well as immunosuppression.

In conclusion, this study demonstrated that the ASFV HLJ/18 strain has a wide range of tissue tropism, causing severe histopathological damage in multiple organs characterized by apoptosis and hemorrhage, which ultimately lead to multi-organ failure and the death of the infected pig. The results provide scientific data in the pathological diagnosis and pathogenic mechanism of ASFV.
